# Case of Poisoning by Tinc

**Published:** 1861-12

**Authors:** William Noble

**Affiliations:** Albion, N. Y.


ART. III.—*Case of Poisoning by Tinc. Aconite, by* WILLIAM NOBLE, M. D., *Albion, N. Y.*

I find recorded so few cases of poisoning by Aconite that I presume the following case will not be uninteresting; I therefore copy it from minutes made at the time of its occurrence. On May 25th, 1858 Mrs. S——, one of our most respectable ladies, aged about 57, after pretty active exercise in fixing and moving furniture, during the forenoon, about 12 M., poured from a bottle containing a pint or more of strong Tinc. of Aconite, two, or two and a half ounces, and drank it, supposing it to be currant wine. I saw her in probably forty minutes; she had swallowed two or three ounces of vinegar, vomited freely, and continued to after my arrival. I found her with face flushed and distorted, as if in agony, labored asthmatic breathing with great groaning at each respiration; complained of a choking and suffocating sensation; extremities cold, no pulse in the wrist or carotids, or any perceptible action of the heart; could swallow but with much difficulty, frequent retching and spitting a tenacious slimy sputa, with spasms intermitting from three to five minutes, when a most violent one took place in which the teeth were fixed, eyes set, voice lost, and to all appearance dying. This passed off, and they became lighter, voice and sight returned, which last had been absent since before I came in; then followed for a short time hydrophobic spasms, which prevented her taking anything. Her pains she described as "AGONY!" "Oh, such agony!" but said her pain was not acute. "Here, "here," she would say, clutching her chest and throat, "I must have something to stop this choking or I shall die," &c., &c. The numbness she declared was all over her; the power over the muscular contractions was very little interrupted, save during the spasms; senses good all the time. At 2.45 P. M. a spasm took place in which she died. She took a small quantity of alcohol, camphor and water, and hot tea, which she begged for—externally constant frictions, mustard sinapisms, hot foot baths and horseradish leaves, which produced warmth once or twice, but it could not be retained; they served to keep respiration up, but no re-action of the heart.

As to the strength of the aconite, it was prepared by A. I Mathews & Co, for a gentleman who used it as an external application for neuralgia, and from using it I believe it as strong as any tinc. made of the root, a drop producing numbness of the tongue, and its peculiar effect for a long time upon the mouth. If any treatment could give the slightest hope in such a case, I would be glad to have it made known.

